# Pathophysiology of Ischemic Stroke: Noncoding RNA Role in Oxidative Stress

**DOI:** 10.1155/2022/5815843

**Published:** 2022-09-12

**Authors:** Zhongzhou Su, Yingze Ye, Chengen Shen, Sheng Qiu, Yao Sun, Siping Hu, Xiaoxing Xiong, Yuntao Li, Liqin Li, Hongfa Wang

**Affiliations:** ^1^Department of Neurosurgery, The Affiliated Huzhou Hospital, Zhejiang University School of Medicine (Huzhou Central Hospital), Huzhou, China; ^2^Huzhou Key Laboratory of Basic Research and Clinical Translation for Neuro Modulation, Huzhou, China; ^3^Department of Neurosurgery, Renmin Hospital of Wuhan University, Wuhan, China; ^4^Department of Anesthesiology, The Affiliated Huzhou Hospital, Zhejiang University School of Medicine (Huzhou Central Hospital), Huzhou, China; ^5^Rehabilitation Medicine Center, Department of Anesthesiology, Zhejiang Provincial People's Hospital, Affiliated People's Hospital, Hangzhou Medical College, Hangzhou, China

## Abstract

Stroke is a neurological disease that causes significant disability and death worldwide. Ischemic stroke accounts for 75% of all strokes. The pathophysiological processes underlying ischemic stroke include oxidative stress, the toxicity of excitatory amino acids, ion disorder, enhanced apoptosis, and inflammation. Noncoding RNAs (ncRNAs) may have a vital role in regulating the pathophysiological processes of ischemic stroke, as confirmed by the altered expression of ncRNAs in blood samples from acute ischemic stroke patients, animal models, and oxygen-glucose-deprived (OGD) cell models. Due to specific changes in expression, ncRNAs can potentially be biomarkers for the diagnosis, treatment, and prognosis of ischemic stroke. As an important brain cell component, glial cells mediate the occurrence and progression of oxidative stress after ischemic stroke, and ncRNAs are an irreplaceable part of this mechanism. This review highlights the impact of ncRNAs in the oxidative stress process of ischemic stroke. It focuses on specific ncRNAs that underlie the pathophysiology of ischemic stroke and have potential as diagnostic biomarkers and therapeutic targets.

## 1. Introduction

Globally, stroke is the cause of the second-highest deaths and the most disability-adjusted life years (DALYs). Stroke is a significant economic burden and stress on society worldwide [1, 2]. Nearly 60% of all strokes occur in people under 70 years old, and stroke incidence rates have shown a sharp and steady increase among young people aged 15 to 49 years [3]. Strokes can be classified into hemorrhagic or ischemic strokes, and the latter accounts for nearly 87% of all stroke cases [4]. A cerebral artery embolism leads to ischemic stroke with ischemia and hypoxia in the infraction of the corresponding brain areas, resulting in neuronal death and irreversible neurological deficits. After ischemia, neurons can immediately not maintain their normal transmembrane ion gradient and homeostasis. This triggers several processes that lead to cell death, such as excitotoxicity, oxidative and nitratative stress, inflammation, and apoptosis. These pathophysiological processes are highly detrimental to neurons, glial, and endothelial cells [5–7]. They are interrelated and continuously trigger each other in a positive feedback loop that destroys neurons [8]. Furthermore, ischemia-reperfusion injury (IRI) that occurs once blood flow is restored may exacerbate these processes [9]. During rapid blood flow recanalization, the demand for sugars and oxygen increases rapidly, oxidase is activated in large amounts, and the degree of tissue oxidation increases greatly. These changes result in a cellular “oxidative burst” and excessive formation of reactive oxygen species (ROS), leading to secondary cerebral ischemia and reperfusion brain damage. As the most basic and critical pathological progression of brain injury, oxidative stress causes neuronal apoptosis, activation of inflammatory signaling pathways, and impairment of the blood-brain barrier (BBB) [10–12].

ncRNAs are a class of functional RNAs. While they cannot code for proteins, ncRNAs regulate gene expression in a posttranscriptional manner, including microRNAs (miRNAs), long noncoding RNAs (lncRNAs), and circular RNAs (circRNAs) [13, 14]. NcRNAs have been reported to be abundantly expressed in the mammalian brain. Additionally, recent studies have depicted that cerebral ischemia alters the expression profiles of ncRNAs [15, 16]. According to many studies, ncRNAs are involved in oxidative stress by controlling transcription and translation, thereby affecting neuronal cell survival [17–19].

Despite the unfavorable results of clinical trials, preclinical studies have suggested that oxidative stress damage may be a potential therapeutic target in ischemic stroke. Dysregulation of ncRNAs is a known mechanism contributing to cerebral ischemia, and potential biomarkers and therapeutic targets for treating cerebral ischemia have been identified. However, none of these breakthroughs have been successfully implemented in clinical practice. This review is aimed at discussing the role of noncoding RNA in oxidative stress in postischemic stroke brain injuries to lay the foundation for therapy and prophylaxis.

## 2. Oxidative Stress in Ischemic Stroke and Ischemia-Reperfusion Injury

The brain accounts for 20% of the total oxygen consumption. Accordingly, it has poor tolerance to hypoxia. When blood flow is interrupted, the ischemic area of the brain cannot maintain redox homeostasis and ion balance due to the lack of oxygen and glucose, which affects cell electrochemistry, metabolism, and the release of toxic products. Anoxic depolarization and various processes are triggered by the massive efflux of K^+^ and influx of Na^+^, water, and Ca^2+^. These result in oxidative and nitrosative stress, excitotoxicity, inflammation, and apoptosis, eventually injuring neurons, glia, and endothelial cells [5, 7, 20–22]. During this process, numerous free radicals are formed, including reactive oxygen species (ROS) and reactive nitrogen species (RNS), which participate in the breakdown of antioxidant systems and lead to brain damage caused by ischemic stroke as well as cerebral ischemia-reperfusion injury [23]. However, two phases of ischemia and reperfusion have differences with regard to the source of free radicals and state of oxidative stress.

### 2.1. Oxidative Stress in the Phases of Ischemia

During the ischemic period, restricted oxygen availability is associated with acidosis, energy deficiency, and changes in ion homeostasis, leading to compensatory brain dysfunction and eventually neuronal death [24, 25]. In the presence of residual oxygen, e.g., in low-flow ischemia, ROS is produced mainly in mitochondria. Under physiological conditions, superoxide dismutase (SOD), glutathione peroxidase (GPX), catalase, and other antioxidant enzymes can aid in maintaining a neutral balance and catalytically protect brain tissues from the cytotoxicity of reactive oxygen species [26]. In addition, ROS play a physiological role by regulating immune system function, maintaining redox homeostasis, and participating in various metabolic pathways, even as second messengers [27, 28]. Endothelial cells rich in mitochondria are efficient sources of ROS. Due to their inherent characteristics and environmental factors, they are especially vulnerable to oxidative stress-induced damage. The effects of ROS include excessive lipid peroxidation and alterations in the functions of receptors, ion channels, and other membrane proteins, subsequently affecting the fluidity and permeability of cell membranes [29–31]. These pathologies cause damage to the blood-brain barrier (BBB) and often lead to leukocyte infiltration and edema [32, 33]. Furthermore, neuronal function relies on the continuous availability of ATP. As ischemic stroke depletes oxygen in the brain, neurons can no longer maintain their transmembrane gradient, and neuronal signaling is impaired [34]. In addition, glucose and oxygen deprivation inhibits ATP synthesis and blocks Na/K-ATPase activity. As a result, calcium ions flow into the cell [29–31]. Increased Ca^2+^ concentration activates cyclases (cox-1 and cox-2) and phospholipase A2, which not only increases ROS production but also enhances glutaminergic neurotransmission [32, 35]. Increased levels of sodium, calcium, and adenosine diphosphate (ADP) also contribute to the overproduction of mitochondrial ROS (mROS) [36, 37]. inducing neuron apoptosis and death [38, 39].

Nitric oxide (NO) is another substance that promotes oxidative stress. NO peaks rapidly at 0.5 h after MCAO and immediately decreases to a low level at 4 h together with eNOS/Nnos. Then, NO gradually increased with the increase in iNOS and peaked at 46 h [40]. In studies of ischemic stroke patients, increases in NO metabolites from day 1 to day 2 were beneficial for neurological function, while sharp increases in NO metabolites from days 2 to 7 were associated with a doubling of infarct volume [40]. NO displays cytotoxicity by destroying cellular DNA, blocking mitochondrial activity, and enhancing nitrifying damage by forming peroxynitrite (ONOO^−^) [41, 42]. NO is usually produced by endothelial nitric oxide synthase (eNOS). However, under inflammatory conditions, smooth muscle cells and macrophages overexpress inducible nitric oxide synthase (iNOS), thus producing large amounts of NO [43]. Moreover, NO is also produced by neuronal nitric oxide synthase (nNOS) [32, 40]. When it collides with NO, both molecules react quickly to form highly reactive ONOO^−^[44]. Superoxide anions can also be dismutated into the more stable H_2_O_2_ through a reaction catalyzed by superoxide dismutase (SOD). In the central nervous system, O_2_^−^ is one of the most important reactive oxygen species as it damages ROS-producing cells and neighboring cells [43, 45]. Superoxide, a byproduct of mitochondrial respiratory chain reactions, is the product of xanthine oxidase (XO) and nicotinamide adenine dinucleotide phosphate (NADPH) oxidase (NOX) activities [43, 46–48]. Interestingly, as the levels of superoxide increase, the NO radical has a dual effect. To be specific, NO interferes with SOD by reducing the antioxidant effect of SOD [43].

### 2.2. Oxidative Stress in the Phases of Reperfusion

The reperfusion of ischemic tissue has long been thought to be beneficial for tissue injury recovery. However, in the 1970s, reports of paradoxical enhancement of the injury response after ischemic (or hypoxic) tissue reperfusion (or reoxygenation) appeared, and the assumed beneficial effects of early reperfusion on tissue recovery after ischemia were questioned [49]. The question was solved when it was first found that the sudden resupply of molecular oxygen to energy- (and oxygen-) deficient tissues resulted in a special injury response not seen in hypoxic stress [50]. The discovery of this reoxygenation-dependent injury response, now known as “reperfusion injury,” established a new field of scientific research that has since grown rapidly and continuously.

**Figure 1:**
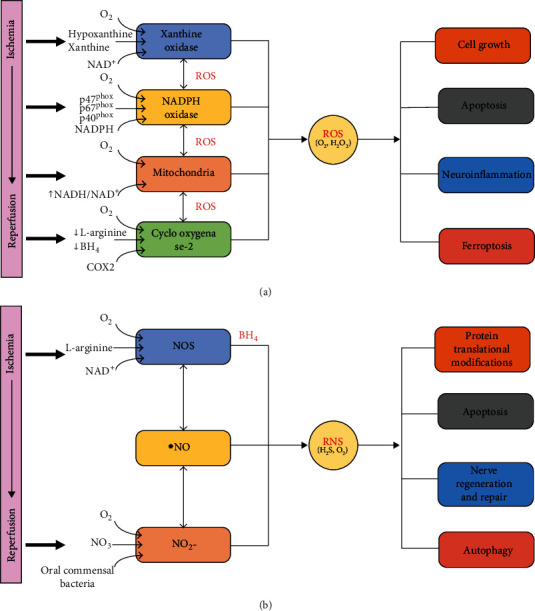
The sources of ROS and RNS during cerebral ischemia-reperfusion injury. During reperfusion, the overproduction of ROS originates from four pathways: mitochondrial respiratory chain, cyclooxygenase-2-catalyzed arachidonic acid reaction, NADPH oxidase, and xanthine and hypoxanthine via xanthine oxidase. In addition, NO and ONOO^−^ are two common types of RNS that have been frequently reported in cerebral ischemia-reperfusion injury.

During reperfusion, the overproduction of ROS originates from four pathways: mitochondrial respiratory chain, cyclooxygenase-2-catalyzed arachidonic acid reaction, NADPH oxidase, and xanthine and hypoxanthine via xanthine oxidase (Figure 1). In the stage of early reperfusion, when microglia and other peripheral immune cells infiltrate, activation of NADPH oxidase in these immune cells contributes to the production of ROS, a phenomenon known as the “oxygen burst.” NADPH oxidase also produces ROS in other cells, such as vascular endothelial cells [51]. When the blood flow is reinstated, a large amount of oxygen arrives and accelerates oxidative damage. Furthermore, oxidative stress during ischemia and reperfusion is known to activate proapoptotic signaling pathways such as cytochrome c signaling, induce DNA damage, alter protein structure and function, and induce lipid peroxidation [52–54]. In addition, oxidative stress can directly regulate important molecules in cellular signaling circuits, such as ion channels and protein kinases [55]. Over the past 25 years, researchers have found that hydrogen peroxide and possibly superoxide play a physiological role in cell signaling and transcriptional regulation [56]. Later research revealed that hydrogen peroxide is also produced under physiological conditions, e.g., in response to growth signals, and it can be overproduced in transformed cells expressing oncogenic mutant Ras [57]. ROS is produced in response to various ligands, including growth factors, cytokines, and G protein-coupled receptors [58, 59]. Therefore, during the recovery phase of reperfusion, low ROS concentrations play a key role in biotransduction signaling, which may be an important reason for promoting recovery from brain tissue damage during the recovery phase.

As displayed in Figure 1, NO and ONOO^−^ are two common types of RNS frequently reported in cerebral ischemia-reperfusion injury. Low levels of NO, produced by endothelial nitric oxide synthase, have physiological functions; conversely, high levels of NO, produced by inducible nitric oxide synthase (NOS) and neuronal nitric oxide synthase (nNOS), have effects on ischemic brain tissue. iNOS and nNOS are known to lead to inflammation, cell death, increased blood-brain barrier permeability, and increased infarct size. During cerebral ischemia or cerebral ischemia-reperfusion injury, NO is produced simultaneously with superoxide anion (O^2-^) and rapidly reacts with O^2-^ at a diffusion-limited rate to generate ONOO^−^. Peroxynitrite readily penetrates the lipid bilayer. It then impairs cell signaling by causing lipid peroxidation of the membrane, mediates nitration of tyrosine residues, and inhibits tyrosine phosphorylation. Peroxynitrite inactivates aconitase and superoxide dismutase, mediates NO-induced BBB damage, and triggers apoptotic cell death (Figure 1).

## 3. Roles of ncRNAs in Ischemia Stroke-Induced Oxidative Stress

### 3.1. miRNA Involved in Oxidative Stress following Ischemia Stroke

**Figure 2:**
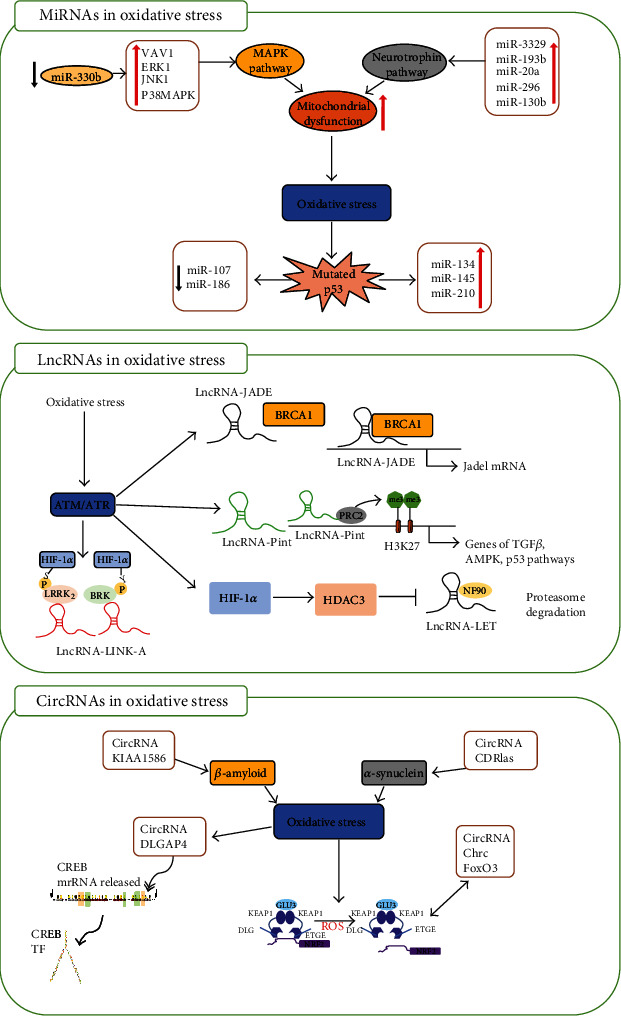
A mechanistic diagram of representative ncRNAs involved in oxidative stress under cerebral ischemia-reperfusion injury. Three different types of ncRNAs are involved in the mechanism of oxidative stress regulation in ischemic stroke, including oxidative stress induction, regulation, and effect. The ncRNAs in figure are taken as examples.

miRNAs, small noncoding RNA superfamily members, are endogenous single-stranded RNA molecules of about 18–25 nucleotides [60]. They act as negative regulators for more than 60% of protein-coding gene expressions by degrading or translationally inhibiting target mRNAs [61–63]. miRNAs can simultaneously modulate targets involved in the pathophysiological process of cerebral ischemia. Therefore, they are considered to have potential as diagnostic and prognostic biomarkers and promising therapeutics in treating ischemic stroke [64, 65]. miRNAs are produced as long primary transcripts (prior-miRNAs) and cleaved by Drosha RNase III endonuclease to result in dry ring intermediates (pre-miRNAs) of approximately 60 to 70 nucleotides [66]. The pre-miRNAs are then exported from the nucleus to the cytoplasm, where they are treated by Dicer RNase II endonucleases to form mature miRNAs of approximately 22–25 nucleotides [67]. Next, the mature miRNAs bind to multiprotein complexes called RNA-induced silencing complexes (RISC), which then bind to the 3′-untranslated region (UTR) of their respective target mRNAs to inhibit translation [68]. Previous studies have found that miRNAs can be potential targets and modulators of oxidative stress-related pathways [69]. miRNAs associated with oxidative stress-related pathways are known as oxidative stress-responsive miRNAs [70]. Intracellular ROS can inhibit or promote miRNA expression and thus produce subsequent biological effects by regulating their direct target genes [71] (Figure 2).

The transient expression of miRNA was observed in blood and brain samples in the MCAO model after reperfusion. Additionally, miR-124a and -290 were upregulated after IR, targeted VSNL1 [72], encoded neuronal calcium sensor proteins in cerebellar granulosa cells, and regulated intracellular signaling pathways directly or indirectly by regulating cyclic nucleotide and MAPK pathways [73], therefore playing an active role in cell death, migration, and neuronal plasticity under pathological conditions such as stroke [74, 75]. Furthermore, miR30a-3p, -99a, -99b, -100, -223, and -383 were upregulated after IR and targeted AQP4 [72], which was speculated to reduce cerebral edema due to its action as a water-selective channel in the plasma membrane of many cells and maintains cerebral hydrohomeostasis [76, 77]. miR-132 and -664 were downregulated after IR and targeted MMP9 [72], which disrupted the blood-brain barrier and caused cerebral edema. In addition, serum MMP-9 level was noted to be correlated with the severity of clinical stroke [78–80].

Based on transcriptome analysis, mitochondrial dysfunction and increased oxidative stress were the molecular mechanisms of miR-210 blockade, leading to increased tissue damage. While miR-210 can alleviate the decreased oxidative metabolism caused by tissue hypoxia, miR-210 also increases the accumulation of ROS, causing cell death and tissue damage [81–83]. However, in ischemic rats, the neuroprotective effects of decreased apoptosis and antioxidant stress response to vagus stimulation were associated with increased miR-210 expression. Protection decreased when miR-210 was blocked, thus suggesting that miR-210 is a neuroprotective factor against ischemia/reperfusion injury [84].

miR-124 is preferentially expressed in the cerebral cortex and cerebellum, initially at low levels in neural progenitors and subsequently at high levels in differentiated and mature neurons [85, 86]. In addition, miR-124 has been reported to protect PC12 cells from OGD/R-induced apoptosis by reducing oxidative stress via the PI3K/AKT/Nrf2 pathway [87]. Moreover, miR-124 enhances neurological recovery in various neurological illnesses by reducing oxidative stress after spinal cord damage via Bax [88, 89]. miR-124 inhibits inflammatory activation under oxidative stress and thereby delays the progression of Alzheimer's disease (AD) [90]. These findings suggest that miR-124 is an important therapeutic target for inhibiting oxidative stress in ischemic stroke.

miR-217 is highly expressed in MCAO rats and the OGD cell model, and its expression level positively correlates with cognitive impairment in MCAO rats. miR-217 also deregulates MEF2D, regulates HDAC5 and ND6 expression, and promotes mitochondrial ROS production, thus leading to enhanced neuronal damage in ischemic stroke and IR [91].

### 3.2. lncRNAs Involved in Oxidative Stress following Ischemia Stroke

lncRNAs of more than 200 nucleotides are cell- and tissue-specific. These may be classified based on the genomic placement between the coding areas of their functional genes (long intergenic ncRNAs) or by coding gene overlap in either the consensus or antisense direction [92, 93]. As guides for chromatin modification complexes or transcription factors in the nucleus, cytoplasmic lncRNAs typically regulate mRNA translation by acting as competing endogenous RNA (ceRNA) or controlling mRNA stability [94]. Also, the lncRNA expression profile in the ischemic penumbra was significantly altered after 1 h reperfusion in MCAO rats [95] (Figure 2).

In patients with ischemic stroke, lncRNA ZFAS1 is significantly downregulated [96, 97]. However, upregulated lncRNA-ZFAS1 can ameliorate brain injury in MCAO rats. It directly sponges miR-582 by promoting NOS3 expression and attenuating I/R-induced inflammation and cell apoptosis via oxidative stress. Studies have also mentioned that lncRNA-ZFAS1 can scavenge miR-186-5p by increasing the expression of the apoptosis regulator MCL1 and rescuing OGD-induced apoptosis of N2a cells [98].

lncRNA-H19 is a maternally derived gene on human chromosome 11. It is associated with stroke susceptibility in the Chinese population [99]. A previous study found that lncRNA-H19 was upregulated within 3 h after stroke, whereas levels of lncRNA-H19 were positively correlated with NIHSS scores of stroke patients within 3 h after stroke onset [100]. It demonstrated antioxidant capacity in metformin-mediated neuronal protection in ischemic stroke [101]. In the OGD/R model, inhibited lncRNA-H19 could reverse metformin-mediated SOD accumulation and MDA elimination [101]. This function was enabled by direct targeting of miR-148a-3p to regulate Rock2/HO-1/Nrf2 [101]. lncRNA-H19 also inhibited miR-19a and upregulated the inhibitor of DNA binding/differentiation 2 (Id2) that led to neuronal apoptosis induced by hypoxia [100].

lncRNA-SNHG14, also known as UBE3A-ATS, is an inhibitor of UBE3A, a brain-specific gene associated with neuronal development. It is involved in neuroinflammation after stroke [102]. lncRNA-SNHG14 promotes the accumulation of NO in microglia, leading to continuous activation of microglia, which causes apoptosis of neurons via the miR-145-5p/PLA2G4A axis [102]. Moreover, inhibition of lncRNA-SNHG14 SOD inhibits MDA accumulation and degradation in the BV-2 OGD model by regulating the miR-199b/AQP4 axis [103].

In lncRNA PVT1-inhibited MCAO rats, oxidative stress and neuron apoptosis were limited, and neurological impairments improved. In MCAO rats, the lncRNA PVT1 was activated by the sex-determining region Y-box 2 (SOX), sponged miR-24-3p, and regulated STAT3 expression [104].

In the SH-SY5Y OGD/R model, lncRNA SNHG15 was highly expressed and promoted the activation of oxidative stress signaling pathways by directly targeting miR-141/SIRT1 [105]. Moreover, miR-183-5p reversed lncRNA SNHG15-induced ROS accumulation in OGD/R-treated SH-SY5Y by directly targeting FOXO1 [106]. Furthermore, lncRNA SNHG15 retention reduced ROS accumulation in PC12 cells treated with OGD/R via the miR-455-3p/TP53INP1 axis [107].

Next, lncRNA OIP5-AS1 was downregulated in ischemic stroke patients, MCAO/R rats, and OGD/R-treated BV2 cells. Overexpression of OIP5-AS1 significantly decreased MDA accumulation, GSH, and overconsumption of SOD, thus counteracting neuroinflammation and oxidative stress and protecting neuronal injury by activating CTRP3 via sponging of miR-186-5p [108].

### 3.3. circRNA Involves in Oxidative Stress after Ischemia Stroke

circRNAs (single-stranded and conserved RNA molecules) are formed by the cleavage of many primary RNA transcripts that synthesize mRNA [109]. As they lack a well-defined 50- and 30-terminus [110], circRNAs can remain stable under the stress of RNase. circRNA regulates gene expression by various mechanisms, including functioning as a cornea through spongy miRNAs, forming ternary complexes with proteins, and encoding proteins [111–113]. circRNAs are abundant in brain tissue and involved in the development of vascular disease. Accordingly, they have been associated with neurological function [114] and acute ischemic stroke [115] (Figure 2).

Based on gene sequencing and KEGG analysis, circRyr2_23, circGucy1a2_7, circCamta1_9, circSmad4_4, and circDlgap3_1 play important roles in regulating oxidative stress by accessing Hif-1, Nrf, and VEGF signaling pathways [116]. The downregulation of circGucy1a2_7 and circRyr2_36 that occurs after stroke should spontaneously resist oxidative stress by adsorbing miR-7a5p to regulate Keap1/NRF-2 signaling [116–118].

circCCDC9 was downregulated in MCAO mice and remained at a low level for 72 h [119]. The upregulated circCCDC9 could restore eNOS expression, reduce oxidative stress, and protect the blood-brain barrier [119–121].

In patients with acute cerebral ischemic stroke, blood levels of circPHKA2 were downregulated. The same results were noted in human brain microvascular endothelial cells (HBMECs) treated with OGD [122]. Further studies confirmed that the upregulation of circPHKA2 decreased the accumulation of ROS and MDA, as well as increased SOD and GSH in OGD-HBMECs, which was due to the competitive binding of miR-574-5p to modulate SOD2 [122].

Various studies on microarrays and sequencing analyses have reported abnormal circRNA expression in ischemic stroke. Furthermore, analyses of Gene Ontology (GO) and Kyoto Encyclopaedia of Genes and Genomes (KEGG) recalled these circRNAs to be prominent in neuroinflammation, apoptosis, and oxidative stress [123]. However, a few studies have explained the mechanism of circRNA involvement in oxidative stress after ischemic stroke, which has also been explored in other diseases [124] (Table 1).

## 4. Noncoding RNA Therapy for Ischemic Stroke

It is extremely important to find objective and effective biomarkers for stroke, because these indicators can not only help the early diagnosis and prognosis assessment of stroke but also serve as therapeutic targets to assist the development of new drugs. Universality, stroke can cause cascade changes of chemicals and transcripts in brain tissue, while conserved expression, defined specificity, high stability, and abundance are the main characteristics of ncRNAs that render them very attractive diagnostic tools for assessing diseases. Thus far, studies have demonstrated the potential of ncRNAs as a cancer diagnostic marker, and recently, studies have shown that ncRNA is also a potential diagnostic marker for neurodegenerative diseases [92, 125, 126].

In the past few years, several in vivo and in vitro studies have demonstrated that certain ncRNAs change over time after ischemic stroke, and they are expected to be widely used as biomarkers in clinical practice. The observed variations in ncRNA amounts in blood samples could be helpful biomarkers that reflect the pathophysiological state of the brain, thus implying that circulating ncRNAs have potential prospects. In addition, Dykstra-Aiello et al. [127] discovered aberrant ncRNA expression in peripheral blood of stroke patients with sex differences, suggesting that certain ncRNAs may be useful biomarkers for stroke development. Wang et al. [128] investigated patients with ischemic stroke and reported that mutations in the H19 gene increased the risk of ischemic stroke. Another independent study revealed that lncRNA H19 levels were significantly increased in the blood and cytoplasm of stroke patients, with high diagnostic sensitivity and specificity levels. This indicates that lncRNA H19 may be a novel diagnostic and therapeutic target for ischemic stroke. Moreover, Mehta et al. [129] suggested that lncRNA FosDT could reduce the loss of motor function after cerebral infarction and stroke via the regulation of REST downstream genes.

In clinical practice, noncoding RNAs are abnormally expressed in the blood of ischemic stroke patients and are closely related to patient prognosis. In whole blood, studies have found that miR-122, miR-148a, let-7i, miR-19a, miR-320d, and miR-4429 are downregulated, while miR-363 and miR-487b are upregulated [130]. Moreover, Lu et al. [131] put forward the idea of noncoding RNAs as potential clinical biomarkers for disorders in the CNS. They suggest that noncoding RNAs can regulate CNS function and many diseases and can be used as a potential biomarker for the diagnosis and prognosis of CNS diseases, as well as combined with other biomarkers and imaging tools to improve the diagnostic power. Subsequently, Mehta et al. [16] conducted a comprehensive circRNA expression profile analysis on male tMCAO mice. microRNA-binding sites, transcription factor binding and gene ontology of circRNAs altered after ischemia were determined under cerebral ischemia. In their study, a total of 1322 detectable circRNAs were comprehensively analysed, of which 283 had significant changes. Their research shows that these noncoding RNAs altered after stroke may be controlled by a set of transcription factors. These noncoding RNAs are involved in many processes and functions such as biological regulation, metabolism, cell communication, and binding with proteins, ions, and nucleic acids. Liu et al. [132] also studied the expression profile of ncRNAs in ischemic stroke and confirmed that noncoding RNA is a potential target for diagnosis and treatment of stroke.

Current research has discovered the role of certain functional ncRNAs such as lncRNA H19 and MALAT1 in ischemic stroke. However, research on ncRNAs still faces many challenges. For example, it is difficult to study their molecular mechanisms due to the complexity of the various functions of ncRNAs. Furthermore, many ncRNAs are expressed only in primates. Even though a significant part of the molecular mechanism has been identified, there is still a long way to go before it can be implemented in clinical use.

## 5. Conclusion

In this review, the mechanisms of oxidative stress in ischemic stroke and reperfusion injury were discussed, alongside the involvement of ncRNAs in the pathological process. Additionally, the potential of three types of ncRNAs for treating stroke was explored. With advances in clinical and experimental techniques, continued research into ncRNAs and their pathways could likely lead to developing a new treatment for ischemic stroke.

## Figures and Tables

**Table 1:** A list of ncRNAs involved in oxidative stress under cerebral ischemia-reperfusion injury.

ncRNAs	Functions	References
miRNA		
miR-124a	Encoding neuronal calcium sensor proteins in cerebellar granulosa cells	[68, 70]
miR-290	Regulating cyclic nucleotide and MAPK pathways	[69, 71]
miR30a-3p	Migration and neuronal plasticity	[72]
miR-99	Regulates AQP4	[73]
miR-100	Connects the DNA damage response to histone H4 acetylation	[74]
miR-223	Regulates serum MMP-9 level	[75]
miR-383	Regulates AQP4 and causes cerebral edema	[76]
FmiR-132	Involves the blood-brain barrier disruption	[70]
miR-210	Alleviates the decreased oxidative metabolism caused by tissue hypoxia	[79–81]
miR-124	Protects PC12 cells from OGD/R-induced apoptosis by reducing oxidative stress via the PI3K/AKT/Nrf2 pathway	[83–85]
miR-217	Deregulates MEF2D, regulates the expression of HDAC5 and ND6, and promotes mitochondrial ROS production	[89]
lncRNAs		
lncRNA ZFAS1	Downregulated by oxidative stress	[93, 94]
ANRIL	Represses the expression of INK4A-ARF-INK4B	[96]
lncRNA-H19	Reverses metformin-mediated SOD accumulation and MDA elimination	[98, 99]
lncRNA-SNHG14	Promotes accumulation of NO in microglia, leading to continuous activation of microglia	[99, 100]
lncRNA PVT1	Regulated STAT3 expression and activated by the sex-determining region Y-box 2 (SOX)	[101]
lncRNA SNHG15	Reduces ROS accumulation of PC12 cells treated with OGD/R via the miR-455-3p/TP53INP1 axis	[102–104]
lncRNA OIP5-AS1	Protecting neuronal injury by activating CTRP3 via sponging miR-186-5p	[105]
circRNAs		
circCCDC9	Restores eNOS expression, reduces oxidative stress, and protects the blood-brain barrier	[106–108]
circPHKA2	Decreases the accumulation of ROS and MDA and increases SOD by competitive binding miR-574-5p	[109]

## References

[1] Feigin V. L., Nichols E., Alam T. (2019). Global, regional, and national burden of neurological disorders, 1990-2016: a systematic analysis for the Global Burden of Disease Study 2016. *Lancet Neurology*.

[2] Feigin V. L., Abajobir A. A., Abate K. H. (2017). Global, regional, and national burden of neurological disorders during 1990-2015: a systematic analysis for the Global Burden of Disease Study 2015. *Lancet Neurology*.

[3] Feigin V. L. (2019). Anthology of stroke epidemiology in the 20th and 21st centuries: assessing the past, the present, and envisioning the future. *International Journal of Stroke*.

[4] Feigin V. L., Forouzanfar M. H., Krishnamurthi R. (2014). Global and regional burden of stroke during 1990-2010: findings from the Global Burden of Disease Study 2010. *Lancet*.

[5] Besancon E., Guo S., Lok J., Tymianski M., Lo E. H. (2008). Beyond NMDA and AMPA glutamate receptors: emerging mechanisms for ionic imbalance and cell death in stroke. *Trends in Pharmacological Sciences*.

[6] Ouyang Y. B., Voloboueva L. A., Xu L. J., Giffard R. G. (2007). Selective dysfunction of hippocampal CA1 astrocytes contributes to delayed neuronal damage after transient forebrain ischemia. *The Journal of Neuroscience*.

[7] Xu L., Emery J. F., Ouyang Y. B., Voloboueva L. A., Giffard R. G. (2010). Astrocyte targeted overexpression of Hsp72 or SOD2 reduces neuronal vulnerability to forebrain ischemia. *Glia*.

[8] Siesjö B. K. (1992). Pathophysiology and treatment of focal cerebral ischemia: Part II: mechanisms of damage and treatment. *Journal of Neurosurgery*.

[9] Khatri R., McKinney A. M., Swenson B., Janardhan V. (2012). Blood-brain barrier, reperfusion injury, and hemorrhagic transformation in acute ischemic stroke. *Neurology*.

[10] Kahles T., Luedike P., Endres M. (2007). NADPH oxidase plays a central role in blood-brain barrier damage in experimental stroke. *Stroke*.

[11] Kleinschnitz C., Grund H., Wingler K. (2010). Post-stroke inhibition of induced NADPH oxidase type 4 prevents oxidative stress and neurodegeneration. *PLoS Biology*.

[12] Zhang H. J., Xing Y. Q., Jin W., Li D., Wu K., Lu Y. (2015). Effects of curcumin on interleukin-23 and interleukin-17 expression in rat retina after retinal ischemia-reperfusion injury. *International Journal of Clinical and Experimental Pathology*.

[13] Romano G., Veneziano D., Acunzo M., Croce C. M. (2017). Small non-coding RNA and cancer. *Carcinogenesis*.

[14] Slack F. J., Chinnaiyan A. M. (2019). The role of non-coding RNAs in oncology. *Cell*.

[15] Dharap A., Bowen K., Place R., Li L. C., Vemuganti R. (2009). Transient focal ischemia induces extensive temporal changes in rat cerebral microRNAome. *Journal of Cerebral Blood Flow and Metabolism*.

[16] Mehta S. L., Pandi G., Vemuganti R. (2017). Circular RNA expression profiles alter significantly in mouse brain after transient focal ischemia. *Stroke*.

[17] Kim T., Jeon Y. J., Cui R. (2015). Role of MYC-regulated long noncoding RNAs in cell cycle regulation and tumorigenesis. *Journal of the National Cancer Institute*.

[18] Iyengar B. R., Choudhary A., Sarangdhar M. A., Venkatesh K. V., Gadgil C. J., Pillai B. (2014). Non-coding RNA interact to regulate neuronal development and function. *Frontiers in Cellular Neuroscience*.

[19] Memczak S., Jens M., Elefsinioti A. (2013). Circular RNAs are a large class of animal RNAs with regulatory potency. *Nature*.

[20] Woodruff T. M., Thundyil J., Tang S. C., Sobey C. G., Taylor S. M., Arumugam T. V. (2011). Pathophysiology, treatment, and animal and cellular models of human ischemic stroke. *Molecular Neurodegeneration*.

[21] Rama R., García J. C., Schaller B. (2016). Excitotoxicity and oxidative stress in acute stroke. *Ischemic Stroke*.

[22] Li Y., Yang G.-Y., Lapchak P. A., Yang G.-Y. (2017). Pathophysiology of ischemic stroke. *Translational Research in Stroke*.

[23] Sun M. S., Jin H., Sun X. (2018). Free radical damage in ischemia-reperfusion injury: an obstacle in acute ischemic stroke after revascularization therapy. *Oxidative Medicine and Cellular Longevity*.

[24] Levraut J., Iwase H., Shao Z. H., Vanden Hoek T. L., Schumacker P. T. (2003). Cell death during ischemia: relationship to mitochondrial depolarization and ROS generation. *American Journal of Physiology. Heart and Circulatory Physiology*.

[25] Vanden Hoek T. L., Li C., Shao Z., Schumacker P. T., Becker L. B. (1997). Significant levels of oxidants are generated by isolated cardiomyocytes during ischemia prior to reperfusion. *Journal of Molecular and Cellular Cardiology*.

[26] Yoshioka M., Tanaka K., Miyazaki I. (2002). The dopamine agonist cabergoline provides neuroprotection by activation of the glutathione system and scavenging free radicals. *Neuroscience Research*.

[27] Valko M., Leibfritz D., Moncol J., Cronin M. T., Mazur M., Telser J. (2007). Free radicals and antioxidants in normal physiological functions and human disease. *The International Journal of Biochemistry & Cell Biology*.

[28] Wu L., Xiong X., Wu X. (2020). Targeting oxidative stress and inflammation to prevent ischemia-reperfusion injury. *Frontiers in Molecular Neuroscience*.

[29] Heo J. H., Han S. W., Lee S. K. (2005). Free radicals as triggers of brain edema formation after stroke. *Free Radical Biology & Medicine*.

[30] Ye R., Shi M., Liu Q., Chen J. (2016). Redox imbalance and stroke. *Oxidative Medicine and Cellular Longevity*.

[31] de la Monte S. M. (2012). Brain insulin resistance and deficiency as therapeutic targets in Alzheimer's disease. *Current Alzheimer Research*.

[32] Rodrigo R., Libuy M., Feliú F., Hasson D. (2013). Oxidative stress-related biomarkers in essential hypertension and ischemia-reperfusion myocardial damage. *Disease Markers*.

[33] Durbin R. P. (1975). Letter: acid secretion by gastric mucous membrane. *The American Journal of Physiology*.

[34] Obrenovitch T. P., Urenjak J., Richards D. A., Ueda Y., Curzon G., Symon L. (1993). Extracellular neuroactive amino acids in the rat striatum during ischaemia: comparison between penumbral conditions and ischaemia with sustained anoxic depolarisation. *Journal of Neurochemistry*.

[35] Gerreth P., Maciejczyk M., Zalewska A., Gerreth K., Hojan K. (2020). Comprehensive evaluation of the oral health status, salivary gland function, and oxidative stress in the saliva of patients with subacute phase of stroke: a case-control study. *Journal of Clinical Medicine*.

[36] Maciejczyk M., Gerreth P., Zalewska A., Hojan K., Gerreth K. (2020). Salivary gland dysfunction in stroke patients is associated with increased protein glycoxidation and nitrosative stress. *Oxidative Medicine and Cellular Longevity*.

[37] Ramiro L., Simats A., García-Berrocoso T., Montaner J. (2018). Inflammatory molecules might become both biomarkers and therapeutic targets for stroke management. *Therapeutic Advances in Neurological Disorders*.

[38] Jana A., Hogan E. L., Pahan K. (2009). Ceramide and neurodegeneration: susceptibility of neurons and oligodendrocytes to cell damage and death. *Journal of the Neurological Sciences*.

[39] Broughton B. R. S., Reutens D. C., Sobey C. G. (2009). Apoptotic mechanisms after cerebral ischemia. *Stroke*.

[40] Bladowski M., Gawrys J., Gajecki D., Szahidewicz-Krupska E., Sawicz-Bladowska A., Doroszko A. (2020). Role of the platelets and nitric oxide biotransformation in ischemic stroke: a translative review from bench to bedside. *Oxidative Medicine and Cellular Longevity*.

[41] Chen Z. Q., Mou R. T., Feng D. X., Wang Z., Chen G. (2017). The role of nitric oxide in stroke. *Medical Gas Research*.

[42] Adams L., Franco M. C., Estevez A. G. (2015). Reactive nitrogen species in cellular signaling. *Experimental Biology and Medicine (Maywood, N.J.)*.

[43] Cheng Y. C., Sheen J. M., Hu W. L., Hung Y. C. (2017). Polyphenols and oxidative stress in atherosclerosis-related ischemic heart disease and stroke. *Oxidative Medicine and Cellular Longevity*.

[44] Guix F. X., Uribesalgo I., Coma M., Muñoz F. J. (2005). The physiology and pathophysiology of nitric oxide in the brain. *Progress in Neurobiology*.

[45] Pacher P., Beckman J. S., Liaudet L. (2007). Nitric oxide and peroxynitrite in health and disease. *Physiological Reviews*.

[46] Maciejczyk M., Żebrowska E., Chabowski A. (2019). Insulin resistance and oxidative stress in the brain: What's new?. *International Journal of Molecular Sciences*.

[47] Zhang L., Wu J., Duan X. (2016). NADPH oxidase: a potential target for treatment of stroke. *Oxidative Medicine and Cellular Longevity*.

[48] Duan J., Gao S., Tu S., Lenahan C., Shao A., Sheng J. (2021). Pathophysiology and therapeutic potential of NADPH oxidases in ischemic stroke-induced oxidative stress. *Oxidative Medicine and Cellular Longevity*.

[49] Reimer K. A., Lowe J. E., Rasmussen M. M., Jennings R. B. (1977). The wavefront phenomenon of ischemic cell death. 1. Myocardial infarct size vs duration of coronary occlusion in dogs. *Circulation*.

[50] Hearse D. J., Humphrey S. M., Chain E. B. (1973). Abrupt reoxygenation of the anoxic potassium-arrested perfused rat heart: a study of myocardial enzyme release. *Journal of Molecular and Cellular Cardiology*.

[51] Granger D. N., Kvietys P. R. (2015). Reperfusion injury and reactive oxygen species: the evolution of a concept. *Redox Biology*.

[52] Peters O., Back T., Lindauer U. (1998). Increased formation of reactive oxygen species after permanent and reversible middle cerebral artery occlusion in the rat. *Journal of Cerebral Blood Flow and Metabolism*.

[53] Yamato M., Egashira T., Utsumi H. (2003). Application of in vivo ESR spectroscopy to measurement of cerebrovascular ROS generation in stroke. *Free Radical Biology & Medicine*.

[54] Kalogeris T., Baines C. P., Krenz M., Korthuis R. J. (2016). Ischemia/reperfusion. *Comprehensive Physiology*.

[55] Raedschelders K., Ansley D. M., Chen D. D. (2012). The cellular and molecular origin of reactive oxygen species generation during myocardial ischemia and reperfusion. *Pharmacology & Therapeutics*.

[56] Lambeth J. D., Neish A. S. (2014). Nox enzymes and new thinking on reactive oxygen: a double-edged sword revisited. *Annual Review of Pathology*.

[57] Irani K., Xia Y., Zweier J. L. (1997). Mitogenic signaling mediated by oxidants in Ras-transformed fibroblasts. *Science*.

[58] Sundaresan M., Yu Z. X., Ferrans V. J., Irani K., Finkel T. (1995). Requirement for generation of H2O2 for platelet-derived growth factor signal transduction. *Science*.

[59] Ibsen R. L., Yu X. Y. (1989). Establishing cuspid-guided occlusion with bonded porcelain. *Journal of Esthetic Dentistry*.

[60] Cao J., Zhao C., Gong L. (2022). MiR-181 enhances proliferative and migratory potentials of retinal endothelial cells in diabetic retinopathy by targeting KLF6. *Current Eye Research*.

[61] Li G., Morris-Blanco K. C., Lopez M. S. (2018). Impact of microRNAs on ischemic stroke: from pre- to post-disease. *Progress in Neurobiology*.

[62] Bartel D. P. (2004). MicroRNAs: genomics, biogenesis, mechanism, and function. *Cell*.

[63] Flynt A. S., Lai E. C. (2008). Biological principles of microRNA-mediated regulation: shared themes amid diversity. *Nature Reviews. Genetics*.

[64] Lewis B. P., Burge C. B., Bartel D. P. (2005). Conserved seed pairing, often flanked by adenosines, indicates that thousands of human genes are microRNA targets. *Cell*.

[65] Khoshnam S. E., Winlow W., Farbood Y., Moghaddam H. F., Farzaneh M. (2017). Emerging roles of microRNAs in ischemic stroke: as possible therapeutic agents. *Journal of Stroke*.

[66] Lee Y., Ahn C., Han J. (2003). The nuclear RNase III Drosha initiates microRNA processing. *Nature*.

[67] Almeida M. I., Reis R. M., Calin G. A. (2011). MicroRNA history: discovery, recent applications, and next frontiers. *Mutation Research*.

[68] Eulalio A., Huntzinger E., Izaurralde E. (2008). Getting to the root of miRNA-mediated gene silencing. *Cell*.

[69] Engedal N., Žerovnik E., Rudov A. (2018). From oxidative stress damage to pathways, networks, and autophagy via microRNAs. *Oxidative Medicine and Cellular Longevity*.

[70] Wan Y., Cui R., Gu J. (2017). Identification of four oxidative stress-responsive microRNAs, miR-34a-5p, miR-1915-3p, miR-638, and miR-150-3p, in hepatocellular carcinoma. *Oxidative Medicine and Cellular Longevity*.

[71] Bu H., Wedel S., Cavinato M., Jansen-Dürr P. (2017). MicroRNA regulation of oxidative stress-induced cellular senescence. *Oxidative Medicine and Cellular Longevity*.

[72] Jeyaseelan K., Lim K. Y., Armugam A. (2008). MicroRNA expression in the blood and brain of rats subjected to transient focal ischemia by middle cerebral artery occlusion. *Stroke*.

[73] Braunewell K. H., Klein-Szanto A. J. (2009). Visinin-like proteins (VSNLs): interaction partners and emerging functions in signal transduction of a subfamily of neuronal Ca2+ -sensor proteins. *Cell and Tissue Research*.

[74] Olde Loohuis N. F., Kos A., Martens G. J., Van Bokhoven H., Nadif Kasri N., Aschrafi A. (2012). MicroRNA networks direct neuronal development and plasticity. *Cellular and Molecular Life Sciences*.

[75] Lin C. W., Chang L. C., Tseng G. C., Kirkwood C. M., Sibille E. L., Sweet R. A. (2015). VSNL1 co-expression networks in aging include calcium signaling, synaptic plasticity, and Alzheimer's disease pathways, Front. *Frontiers in Psychiatry*.

[76] Guo Q., Sayeed I., Baronne L. M., Hoffman S. W., Guennoun R., Stein D. G. (2006). Progesterone administration modulates AQP4 expression and edema after traumatic brain injury in male rats. *Experimental Neurology*.

[77] Papadopoulos M. C., Verkman A. S. (2007). Aquaporin-4 and brain edema. *Pediatric Nephrology*.

[78] Horstmann S., Kalb P., Koziol J., Gardner H., Wagner S. (2003). Profiles of matrix metalloproteinases, their inhibitors, and laminin in stroke patients: influence of different therapies. *Stroke*.

[79] Montaner J., Alvarez-Sabín J., Molina C. (2001). Matrix metalloproteinase expression after human cardioembolic stroke: temporal profile and relation to neurological impairment. *Stroke*.

[80] Rosell A., Ortega-Aznar A., Alvarez-Sabín J. (2006). Increased brain expression of matrix metalloproteinase-9 after ischemic and hemorrhagic human stroke. *Stroke*.

[81] Zaccagnini G., Martelli F., Fasanaro P. (2004). p66ShcA modulates tissue response to hindlimb ischemia. *Circulation*.

[82] Jiang Y., Li L., Tan X., Liu B., Zhang Y., Li C. (2015). miR-210 mediates vagus nerve stimulation-induced antioxidant stress and anti-apoptosis reactions following cerebral ischemia/reperfusion injury in rats. *Journal of Neurochemistry*.

[83] Hu S., Huang M., Li Z. (2010). MicroRNA-210 as a novel therapy for treatment of ischemic heart disease. *Circulation*.

[84] Fuschi P., Maimone B., Gaetano C., Martelli F. (2019). Noncoding RNAs in the vascular system response to oxidative stress. *Antioxidants & Redox Signaling*.

[85] Deo M., Yu J. Y., Chung K. H., Tippens M., Turner D. L. (2006). Detection of mammalian microRNA expression by in situ hybridization with RNA oligonucleotides. *Developmental Dynamics*.

[86] Åkerblom M., Sachdeva R., Barde I. (2012). MicroRNA-124 is a subventricular zone neuronal fate determinant. *The Journal of Neuroscience*.

[87] Shu K., Zhang Y. (2019). Protodioscin protects PC12 cells against oxygen and glucose deprivation-induced injury through miR-124/AKT/Nrf2 pathway. *Cell Stress & Chaperones*.

[88] Gong G., Gu Y., Zhang Y., Liu W., Li L., Li J. (2019). RETRACTED: tanshinone IIA alleviates oxidative damage after spinal cord injury in vitro and in vivo through up-regulating miR-124. *Life Sciences*.

[89] Xu Z., Zhang K., Wang Q., Zheng Y. (2019). MicroRNA-124 improves functional recovery and suppresses Bax-dependent apoptosis in rats following spinal cord injury. *Molecular Medicine Reports*.

[90] Feng C. Z., Yin J. B., Yang J. J., Cao L. (2017). Regulatory factor X1 depresses ApoE-dependent A*β* uptake by miRNA-124 in microglial response to oxidative stress. *Neuroscience*.

[91] Shi L., Tian Z., Fu Q. (2020). miR-217-regulated MEF2D-HDAC5/ND6 signaling pathway participates in the oxidative stress and inflammatory response after cerebral ischemia. *Brain Research*.

[92] Esteller M. (2011). Non-coding RNAs in human disease. *Nature Reviews. Genetics*.

[93] Wang M., Gan S., Li B., Wang Y. (2020). Long non-coding RNA-ATB attenuates the angiotensin II-induced injury of vascular endothelial cell. *Annals of Clinical and Laboratory Science*.

[94] Li L., Wang M., Mei Z. (2017). lncRNAs HIF1A-AS2 facilitates the up-regulation of HIF-1*α* by sponging to miR-153-3p, whereby promoting angiogenesis in HUVECs in hypoxia. *Biomedicine & Pharmacotherapy*.

[95] Dharap A., Nakka V. P., Vemuganti R. (2012). Effect of focal ischemia on long noncoding RNAs. *Stroke*.

[96] Zhang Y., Zhang Y. (2020). lncRNA ZFAS1 improves neuronal injury and inhibits inflammation, oxidative stress, and apoptosis by sponging miR-582 and upregulating NOS3 expression in cerebral ischemia/reperfusion injury. *Inflammation*.

[97] Wang J., Ruan J., Zhu M. (2019). Predictive value of long noncoding RNA ZFAS1 in patients with ischemic stroke. *Clinical and Experimental Hypertension*.

[98] Shen B., Wang L., Xu Y., Wang H., He S. (2021). Long non-coding RNA ZFAS1 exerts a protective role to alleviate oxygen and glucose deprivation-mediated injury in ischemic stroke cell model through targeting miR-186-5p/MCL1 axis. *Cytotechnology*.

[99] Wan P., Su W., Zhuo Y. (2017). The role of long noncoding RNAs in neurodegenerative diseases. *Molecular Neurobiology*.

[100] Xiao Z., Qiu Y., Lin Y. (2019). Blocking lncRNA H19-miR-19a-Id2 axis attenuates hypoxia/ischemia induced neuronal injury. *Aging (Albany NY)*.

[101] Zeng J., Zhu L., Liu J. (2019). Metformin protects against oxidative stress injury induced by ischemia/reperfusion via regulation of the lncRNA-H19/miR-148a-3p/Rock2 axis. *Oxidative Medicine and Cellular Longevity*.

[102] Qi X., Shao M., Sun H., Shen Y., Meng D., Huo W. (2017). Long non-coding RNA SNHG14 promotes microglia activation by regulating miR-145-5p/PLA2G4A in cerebral infarction. *Neuroscience*.

[103] Zhang G., Li T., Chang X., Xing J. (2021). Long noncoding RNA SNHG14 promotes ischemic brain injury via regulating miR-199b/AQP4 Axis. *Neurochemical Research*.

[104] Chen Z., Fan T., Zhao X., Zhang Z. (2021). Depleting SOX2 improves ischemic stroke via lncRNA PVT1/microRNA-24-3p/STAT3 axis. *Molecular Medicine*.

[105] Kang M., Ji F., Sun X., Liu H., Zhang C. (2021). LncRNA SNHG15 promotes oxidative stress damage to regulate the occurrence and development of cerebral ischemia/reperfusion injury by targeting the miR-141/SIRT1 axis. *Journal of Healthcare Engineering*.

[106] Wen Y., Zhang X., Liu X., Huo Y., Gao Y., Yang Y. (2020). Suppression of lncRNA SNHG15 protects against cerebral ischemia-reperfusion injury by targeting miR-183-5p/FOXO1 axis. *American Journal of Translational Research*.

[107] Fan Y., Wei L., Zhang S. (2021). LncRNA SNHG15 knockdown protects against OGD/R-induced neuron injury by downregulating TP53INP1 expression via binding to miR-455-3p. *Neurochemical Research*.

[108] Chen Y., Liu W., Chen M., Sun Q., Chen H., Li Y. (2021). Up-regulating lncRNA OIP5-AS1 protects neuron injury against cerebral hypoxia-ischemia induced inflammation and oxidative stress in microglia/macrophage through activating CTRP3 via sponging miR-186-5p. *International Immunopharmacology*.

[109] Rybak-Wolf A., Stottmeister C., Glažar P. (2015). Circular RNAs in the mammalian brain are highly abundant, conserved, and dynamically expressed. *Molecular Cell*.

[110] Jeck W. R., Sorrentino J. A., Wang K. (2013). Circular RNAs are abundant, conserved, and associated with ALU repeats. *RNA*.

[111] Huang R., Zhang Y., Han B. (2017). Circular RNA HIPK2 regulates astrocyte activation via cooperation of autophagy and ER stress by targeting MIR124-2HG. *Autophagy*.

[112] Du W. W., Yang W., Liu E., Yang Z., Dhaliwal P., Yang B. B. (2016). Foxo3 circular RNA retards cell cycle progression via forming ternary complexes with p21 and CDK2. *Nucleic Acids Research*.

[113] Yang Y., Gao X., Zhang M. (2018). Novel role of FBXW7 circular RNA in repressing glioma tumorigenesis. *Journal of the National Cancer Institute*.

[114] Kristensen L. S., Andersen M. S., Stagsted L. V. W., Ebbesen K. K., Hansen T. B., Kjems J. (2019). The biogenesis, biology and characterization of circular RNAs. *Nature Reviews. Genetics*.

[115] Song Y. F., Zhao L., Wang B. C. (2020). The circular RNA TLK1 exacerbates myocardial ischemia/reperfusion injury via targeting miR-214/RIPK1 through TNF signaling pathway. *Free Radical Biology & Medicine*.

[116] Wang C., Yang Y., Xu M. (2021). Deep sequencing of the rat MCAO cortexes reveals crucial circRNAs involved in early stroke events and their regulatory networks. *Neural Plasticity*.

[117] Patel M. (2016). Targeting oxidative stress in central nervous system disorders. *Trends in Pharmacological Sciences*.

[118] Kaspar J. W., Niture S. K., Jaiswal A. K. (2009). Nrf2:INrf2 (Keap1) signaling in oxidative stress. *Free Radical Biology & Medicine*.

[119] Wu L., Xu H., Zhang W., Chen Z., Li W., Ke W. (2020). Circular RNA circCCDC9 alleviates ischaemic stroke ischaemia/reperfusion injury via the notch pathway. *Journal of Cellular and Molecular Medicine*.

[120] Zhang Z. H., Wang Y. R., Li F. (2020). Circ-camk4 involved in cerebral ischemia/reperfusion induced neuronal injury. *Scientific Reports*.

[121] Zhang Z., He J., Wang B. (2021). Circular RNA circ_HECTD1 regulates cell injury after cerebral infarction by miR-27a-3p/FSTL1 axis. *Cell Cycle*.

[122] Yang X., Li X., Zhong C. (2021). Circular RNA circPHKA2 relieves OGD-induced human brain microvascular endothelial cell injuries through competitively binding miR-574-5p to modulate SOD2. *Oxidative Medicine and Cellular Longevity*.

[123] Wang Y. Y., Wang Y. Z., Zhang H. Y., He Z. Y. (2021). The role of circular RNAs in brain and stroke. *Frontiers in Bioscience-Landmark*.

[124] Zhang Y., Chen Y., Wan Y. (2021). Circular RNAs in the regulation of oxidative stress. *Frontiers in Pharmacology*.

[125] Rupaimoole R., Slack F. J. (2017). MicroRNA therapeutics: towards a new era for the management of cancer and other diseases. *Nature Reviews. Drug Discovery*.

[126] Matsui M., Corey D. R. (2017). Non-coding RNAs as drug targets. *Nature Reviews. Drug Discovery*.

[127] Dykstra-Aiello C., Jickling G. C., Ander B. P. (2016). Altered expression of long noncoding RNAs in blood after ischemic stroke and proximity to putative stroke risk loci. *Stroke*.

[128] Wang W., Jiang B., Sun H. (2017). Prevalence, incidence, and mortality of stroke in China: results from a nationwide population-based survey of 480 687 adults. *Circulation*.

[129] Mehta S. L., Kim T., Vemuganti R. (2015). Long noncoding RNA FosDT promotes ischemic brain injury by interacting with REST-associated chromatin-modifying proteins. *The Journal of Neuroscience*.

[130] Jickling G. C., Ander B. P., Zhan X., Noblett D., Stamova B., Liu D. (2014). microRNA expression in peripheral blood cells following acute ischemic stroke and their predicted gene targets. *PLoS One*.

[131] Lu D., Xu A. D., Ad X. (2016). Mini review: circular RNAs as potential clinical biomarkers for disorders in the central nervous system. *Frontiers in Genetics*.

[132] Liu C., Zhang C., Yang J. (2017). Screening circular RNA expression patterns following focal cerebral ischemia in mice. *Oncotarget*.

